# Extensive coronavirus-induced membrane rearrangements are not a determinant of pathogenicity

**DOI:** 10.1038/srep27126

**Published:** 2016-06-03

**Authors:** Helena J. Maier, Benjamin W. Neuman, Erica Bickerton, Sarah M. Keep, Hasan Alrashedi, Ross Hall, Paul Britton

**Affiliations:** 1 The Pirbright Institute, Pirbright, Surrey, UK; 2 School of Biological Sciences, University of Reading, Reading, Berkshire, UK

## Abstract

Positive-strand RNA (+RNA) viruses rearrange cellular membranes during replication, possibly in order to concentrate and arrange viral replication machinery for efficient viral RNA synthesis. Our previous work showed that in addition to the conserved coronavirus double membrane vesicles (DMVs), Beau-R, an apathogenic strain of avian *Gammacoronavirus* infectious bronchitis virus (IBV), induces regions of ER that are zippered together and tethered open-necked double membrane spherules that resemble replication organelles induced by other +RNA viruses. Here we compared structures induced by Beau-R with the pathogenic lab strain M41 to determine whether membrane rearrangements are strain dependent. Interestingly, M41 was found to have a low spherule phenotype. We then compared a panel of pathogenic, mild and attenuated IBV strains in *ex vivo* tracheal organ culture (TOC). Although the low spherule phenotype of M41 was conserved in TOCs, each of the other tested IBV strains produced DMVs, zippered ER and spherules. Furthermore, there was a significant correlation for the presence of DMVs with spherules, suggesting that these structures are spatially and temporally linked. Our data indicate that virus induced membrane rearrangements are fundamentally linked to the viral replicative machinery. However, coronavirus replicative apparatus clearly has the plasticity to function in different structural contexts.

All positive-strand RNA (+RNA) viruses studied to date induce the rearrangement of cellular membranes during infection to facilitate viral RNA synthesis and assembly of RNA synthesis machinery into replication-transcription complexes (reviewed in^1^,^2^,^3^,^4^). The types of membrane structures that are induced varies between different families of +RNA viruses but includes single membrane and double membrane vesicles, contiguous vesicles, convoluted membranes (CM), paired membranes and spherules. The source of rearranged membranes also varies between different families of +RNA viruses and can be from intracellular vesicles, endoplasmic reticulum, mitochondria and the plasma membrane. It has long been observed that *Betacoronaviruses* induce the formation of double membrane vesicles (DMVs) during infection of a host cell, along with a reticular network of membranes called convoluted membrane^5^,^6^,^7^,^8^,^9^,^10^,^11^,^12^. During SARS-coronavirus (SARS-CoV) infection, both of these structures were shown to be derived from and linked to cellular ER, as well as linked to each other^10^. More recently, comparable structures were also found in cells infected with *Alphacoronaviruses*^13^. The *Coronavirinae* subfamily is divided into four genera, with an ancestral split giving rise to one progenitor for the *Alpha-* and *Beta-* genera and a second progenitor for the *Gamma-* and *Delta-* genera. Interestingly, in our recent work studying avian *Gammacoronavirus* infectious bronchitis virus (IBV), subtle, but potentially significant, differences in the nature of virus induced membrane rearrangements were reported. Specifically, CM was not observed to any great extent and instead regions of ER were found to become zippered together and tethered to this were pinched out spherules with a neck and channel connecting the spherule interior to the cytoplasm^14^. Consistent with observations from other coronaviruses, IBV infection also induced the formation of DMVs. Although detailed understanding of the function of rearranged membranes in the replication of coronaviruses and numerous other +RNA viruses remains to be determined, the conserved process of membrane rearrangement for the whole virus class, and the close association of replicase proteins and products of RNA synthesis with rearranged membranes strongly supports the hypothesis that they play a key role in viral RNA synthesis. Therefore, further study of these likely critical structures and developing understanding of their role in virus replication will provide insight into this central stage of the virus life cycle.

IBV is a highly important pathogen of poultry, causing animal welfare problems and extensive economic losses to poultry industries globally. There are numerous strains and serotypes of IBV that cause a broad spectrum of disease ranging from mild respiratory symptoms to nephropathology to severe pathology of the reproductive organs^15^,^16^,^17^,^18^. In addition, live-attenuated vaccine strains are commonly used to protect poultry from virus infection (for example Nobilis IB (MSD Animal Health), Poulvac IB (Zoetis) and Cevac (Ceva)). Finally, laboratory adapted strains, capable of replicating in cell culture are used to study viral pathogenesis, the mechanism of virus replication and interaction with the host^19^,^20^. Previous studies investigating the nature of coronavirus induced membrane rearrangements have utilised a limited range of virus strains or have compared wild type and mutant viruses of the same strain to study any involvement of membrane rearrangements in virus replication or viral fitness in cell culture. Indeed, our previous work was performed using an apathogenic lab strain of IBV and recombinants of this virus^14^,^21^. Therefore, we undertook a study to characterise IBV induced membrane rearrangements in more detail to determine whether virus strain, genotype, degree of pathogenicity or the process of virus adaptation to allow replication in cell culture resulted in changes in the nature of membrane rearrangements. Initially, a comparison of apathogenic Beau-R and pathogenic M41, demonstrated that M41 presented a low spherule phenotype, although replication dynamics were comparable. A further panel of pathogenic, apathogenic, lab adapted, field and vaccine strains of IBV was selected and replication and induction of membrane rearrangements was assessed. We found that there were no significant differences in membrane rearrangements induced by the virus panel. Only IBV strain M41 presented a low spherule phenotype.

## Results

### Apathogenic IBV strain Beau-R and pathogenic M41 exhibit comparable replication characteristics in primary chick kidney cells

**Figure 1:**
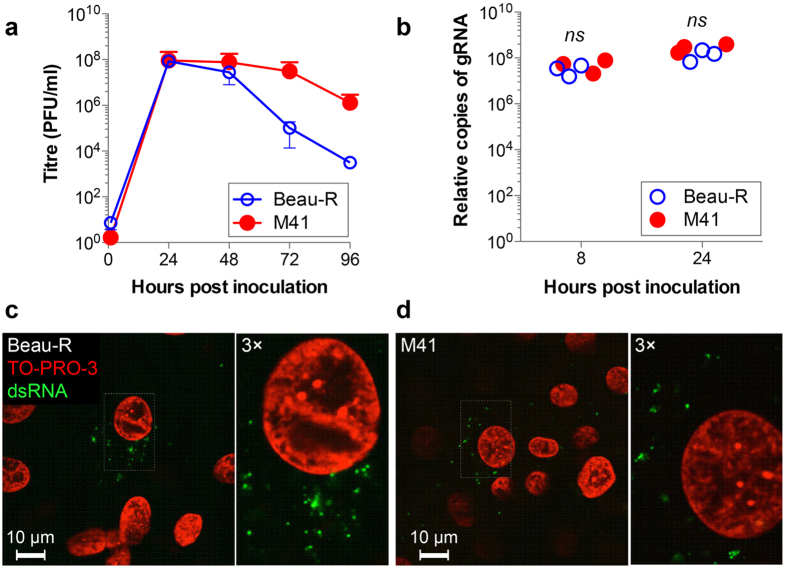
Replication of Beau-R and M41 are comparable in CK cells. (**a**) CK cells were infected with Beau-R (open circles) or M41 (closed circles) at an MOI of 0.005. Release of progeny virus was determined by plaque assay. Mean and standard error of three independent experiments are shown. (**b**) Genomic RNA levels were measured by two-step RT-qPCR at 8 and 24 hours after inoculation of CK cells with 20 pfu per cell of Beau-R or M41. Results from three independent experiments are shown. Non-significant differences by *t*-test are indicated (ns). CK cells were inoculated with (**c**) Beau-R for 4 hours or (**d**) M41 for 3 hours. Cells were fixed with 4% paraformaldehyde and labelled with anti-dsRNA (green) and nuclei were labelled with TO-PRO-3 (red).

In our previous work we characterised the progress of different aspects of replication of apathogenic IBV Beau-R. To begin to understand the relationships between pathogenicity, virus replication and virus induced membrane rearrangements, a comparison was made between Beau-R and M41 infection of primary chick kidney (CK) cells. Initially, viral growth kinetics were analysed. CK cells were infected with either Beau-R or M41 and release of progeny virus assessed over a 96 hour period (Fig. 1a). It can be seen that growth kinetics of both viruses are broadly comparable with a peak of progeny virus release at 24 hours post infection (hpi). It can be noted that the titre of Beau-R decreases after 24 hours to a greater extent than M41, where viral titre plateaus. This may be as a result of the more severe cytopathic effect induced by Beau-R compared to M41. However, both IBV strains are able to replicate to comparable titres within CK cells.

Membrane rearrangements are thought to play an important role in assembly of viral replication complexes, responsible for synthesising viral RNA. Therefore, the total amount of viral genomic RNA (gRNA) was determined in cells infected with either Beau-R or M41. CK cells were infected or mock infected and total cellular RNA harvested at 8 and 24 hpi. The amount of gRNA was then assayed by two-step RT-qPCR using a primer/probe set specific for genome length sequence (Fig. 1b). This analysis showed that the total level of viral gRNA was not significantly different in cells infected with either Beau-R or M41.

Finally, the onset of dsRNA accumulation in infected cells was analysed. Virus associated dsRNA has historically been used as a marker for sites of viral RNA synthesis. Although the reliability of this has increasingly come into question in recent years^10^,^14^,^22^, accumulation of dsRNA still provides an indication of the onset of viral RNA synthesis and the formation of virus induced membrane rearrangements. Therefore, CK cells were infected with Beau-R or M41, fixed at hourly intervals post infection and labelled with an anti-dsRNA antibody. Analysis by confocal microscopy (Fig. 1c–d) showed that dsRNA accumulation during Beau-R infection could be detected in cytoplasmic puncta from 4 hpi, consistent with previous data^14^. By comparison, dsRNA accumulation in cells infected with M41 could be detected earlier, at 3 hpi, again in characteristic cytoplasmic puncta. These data suggest that kinetics of replication of Beau-R and M41 are comparable in primary CK cells, and that M41 may initiate RNA synthesis earlier during infection than Beau-R.

### M41 presents a low spherule phenotype in CK cells

**Figure 2:**
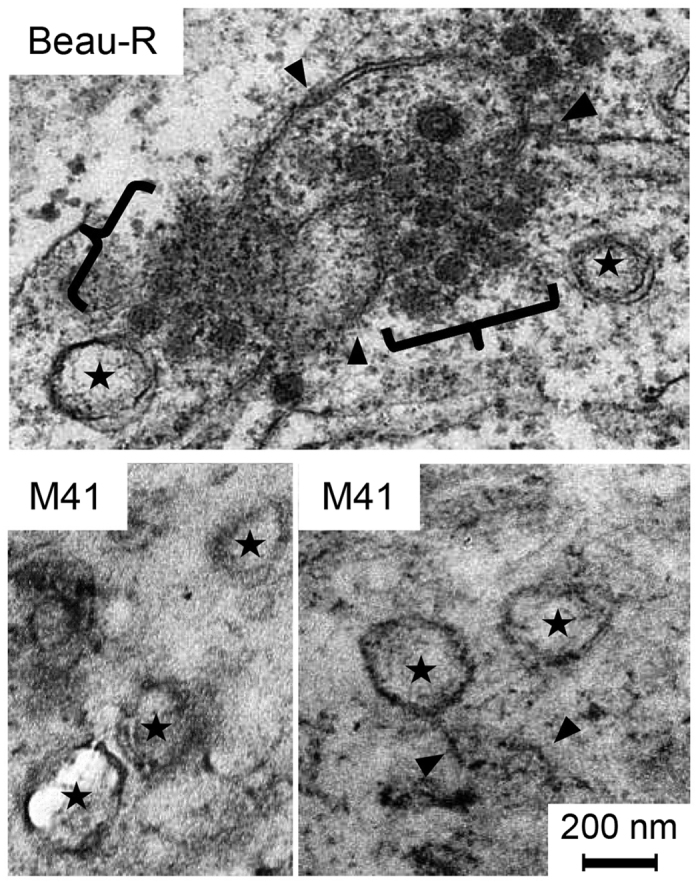
M41 has a low spherule phenotype in CK cells. Primary CK cells were infected with Beau-R or M41. After 24 hours, cells were chemically fixed and visualised by TEM. DMVs are indicated with a star, zippered ER is indicated with a black arrowhead and spherules with black brackets.

To further characterise differences in virus replication and virus-host cell interaction of Beau-R and M41, virus induced membrane rearrangements were analysed. Primary CK cells were infected with either Beau-R or M41, or mock infected, and after 24 hours were fixed and analysed by TEM. In agreement with our previous observations^14^, Beau-R was found to induce the formation of zippered ER and spherules, surrounded by an electron dense region (Fig. 2). In addition, DMVs were identified. In comparison, in CK cells infected with M41, although DMVs and intracellular virus particles were easily identifiable and zippered ER could be recognised, very few spherules were observed (Fig. 2). Indeed, 34 M41 cell sections with visible signs of infection including virions, zippered ER and DMVs were imaged and only 10 spherules were identified, compared to 133 spherules identified from 52 Beau-R infected cell sections. Therefore, in CK cells, M41 exhibits a low spherule phenotype, despite having replication characteristics that were comparable to Beau-R.

### Pathogenic and apathogenic strains of IBV replicate in *ex vivo* tracheal organ cultures

**Figure 3:**
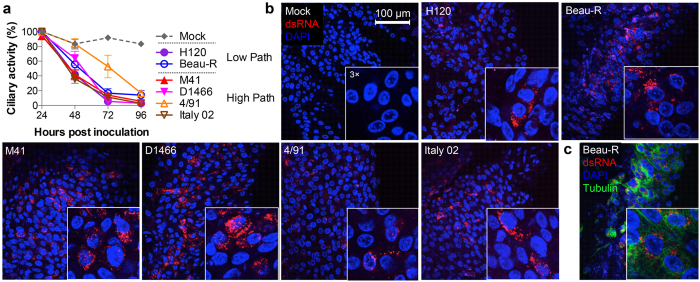
Pathogenic and apathogenic viruses replicate in tracheal organ culture resulting in ciliostasis. (**a**) TOCs were mock infected or infected with H120, Beau-R, M41, D1466, 4/91 or Italy02. Ciliary activity was assessed and scored at 24 hour intervals. The mean and SEM of three independent experiments are shown. (**b**) TOCs were mock infected or infected with H120, Beau-R, M41, D1466, 4/91 or Italy02. After 24 hours, cells were fixed with 4% paraformaldehyde and labelled with anti-dsRNA (red) and anti-tubulin (green, **c**). Nuclei are stained with DAPI (blue).

To determine whether the low spherule phenotype observed for M41 is more broadly associated with IBV pathogenicity in general, the range of pathogenic and apathogenic field and vaccine strains available were utilised. In addition to Beau-R and M41, the selected viral strains were vaccine strain H120 and pathogenic field strains D1466, 4/91 and Italy02 (Table 1). Tracheal organ cultures (TOCs) were found to be useful for the isolation and replication of field isolates of IBV that do not replicate in primary cell culture, as an alternative to embryonated chickens eggs^23^. Replication of IBV within TOCs results in the inhibition of ciliary activity^23^. As a first step to assess whether IBV-induced membrane rearrangements differ according to viral genotype, degree of pathogenicity or laboratory adaptation, ability of the selected viruses to replicate within *ex vivo* tracheal organ cultures (TOCs) was investigated. TOCs were infected with the selected strains of IBV and loss of ciliary activity was measured over a 96 hour period (Fig. 3a). The mock infected TOCs show a fairly stable level of ciliary activity. However, although the overall dynamics seem to alter, TOCs that had been infected with any of the selected strains of IBV show a gradual reduction in ciliary activity over the course of the experiment with complete ciliostasis by 96 hpi.

To further confirm viral replication with TOCs, individual TOCs were mock infected or infected with each virus strain, fixed at 24 hpi and labelled with an anti-dsRNA antibody to visualise infected cells (Fig. 3b) or were labelled with anti-dsRNA and anti-tubulin (Fig. 3c). Confocal microscopy analysis revealed that no virus infection could be detected in the mock infected TOCs. However, virus infection could be detected in all other TOCs. Overall, these data confirmed that all of the selected strains of IBV were capable of infecting and replicating within cells in TOCs, and virus replication resulted in ciliostasis.

### All strains of IBV induce zippered ER, spherules and DMVs

**Figure 4:**
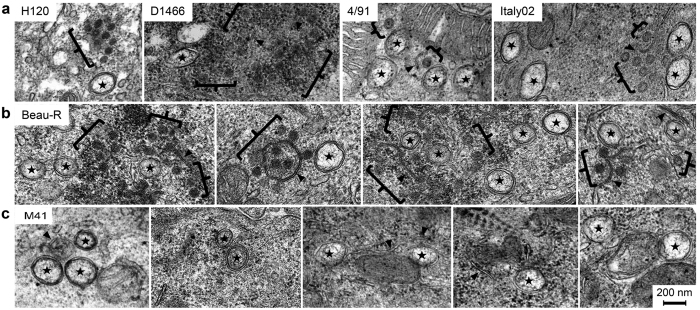
Pathogenic and apathogenic strains of IBV induce zippered ER, spherules and DMVs. TOCs were mock infected or infected with (**a**) H120, D1466, 4/91 or Italy02, (**b**), Beau-R or (**c**) M41. After 24 hours, cells were chemically fixed. DMVs are indicated by a star, zippered ER with black arrowheads and spherules with black brackets.

After confirmation that the selected strains of IBV replicated within TOCs and induced ciliostasis as expected, the ability of each virus to induce membrane rearrangements was assessed. TOCs were infected with each strain of virus and after 24 hours were chemically fixed and analysed by TEM. Again, Beau-R infection was found to result in the formation of DMVs, zippered ER and spherules (Fig. 4). When TOCs were infected with a second apathogenic strain, vaccine strain H120, comparable structures, zippered ER, spherules and DMVs, were all observed. Three pathogenic field strains (D1466, 4/91 and Italy02) also induced zippered ER, spherules and DMVs in infected TOCs. Finally, TOCs infected with the pathogenic laboratory adapted IBV strain, M41, were then assessed. Consistent with earlier observations in CK cells, DMVs, zippered ER and intracellular virus particles were easily detected (see Supplementary Fig. S1) but very few spherules were visible (Fig. 4c). Several examples of images of Beau-R (Fig. 4b) or M41 (Fig. 4c) infected TOCs are shown to demonstrate the absence of spherules in M41 infected cell sections. Therefore, apathogenic, pathogenic, laboratory adapted and field isolates can all induce a full range of membrane rearrangements.

**Figure 5:**
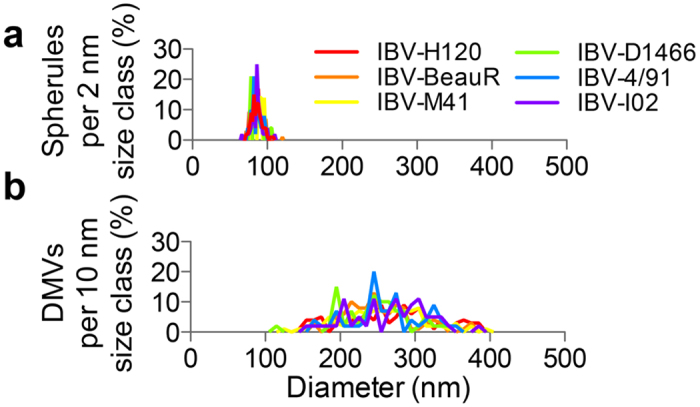
Spherule and DMV diameters are not altered by virus strain. Spherule (**a**) and DMV (**b**) diameters were measured and divided into 2 nm (spherules) or 10 nm (DMV) size classes. The number of spherules or DMVs in each size class is shown for each of the viral strains.

Some physical characteristics of spherules and DMVs were then measured to determine whether the structures induced by the different viral strains were comparable. Firstly, spherule diameters were classed within 2 nm brackets ranging from 50 nm to 110 nm. The percentage of spherules found in cells infected with each IBV strain that fit into these brackets was determined (Fig. 5a). The spread of spherule diameter and the peak diameters were comparable for all IBV strains tested. In addition, DMV diameters were classed within 10 nm brackets from 100 nm to 420 nm. The percentage of DMVs found in cells infected with each IBV strain that fit into each bracket was determined (Fig. 5b). The spread of DMV diameters and the peak DMV diameter were also comparable across all IBV strains. This demonstrates that the size and structure of spherules and DMVs is not altered by viral strain.

Samples were further analysed for the percentage of total cells that presented any signs of infection; either DMVs, spherules or intracellular virus (Table 2). Beau-R showed the highest level of infection with 52% of cell sections showing markers for infection. The remaining viral strains had infection rates of between 23% and 11%. When infection markers were analysed individually, again, Beau-R showed the highest percentage of cell sections containing DMVs, spherules and intracellular virus. The remaining viral strains showed varying levels of presence of infection markers, consistent with the total percentage cell sections that showed any sign of infection. Subsequently, cell sections that had been classified as infected were analysed for the number of DMVs, spherules or intracellular virus. Numbers of DMVs per cell section varied from 2 to 4, number of intracellular virus particles ranged from D1466 with 27 (+/−7) to 4/91 with 11 (+/−4) and finally number of spherules per cell section ranging from 1 to 4, with the exception of M41. M41 infected cell sections only contained an average of 0.1 spherules (+/−0.1). To extend the comparison of Beau-R and M41, images of infected CK cells from Figure 2 were also analysed (Table 2). The percent of CK cell sections showing markers of infection was 37% for Beau-R and 39% for M41. The percentage of cells that showed individual signs of infection were also similar, except for the percentage of M41 infected cells where spherules could be detected, with 2% compared to 14% in Beau-R infected cell sections. Again, the number of different markers of infection found per cell section classified as infected was determined. Indeed, 53 M41 cell sections with visible signs of infection were imaged and only 7 spherules were identified, compared to 248 spherules identified from 69 Beau-R infected cell sections. The most significant observation showing that Beau-R infected cell sections contained an average of 3 spherules (+/−1) whilst M41 infected cell sections contained and average of only 0.3 spherules (+/−0.2). Finally, the percentage of infected TOC cell sections that contained at least 1 DMV, spherule or intracellular virus particle was determined (Table 2). When compared with vaccine strain H120, there were no significant differences in the percentage of infected cells that contained at least 1 DMV or at least 1 virion. Furthermore, the percentage infected cell sections containing at least 1 spherule was not significantly different for any viral strain (range 24–54%) except M41 with only 6% (p = 4 × 10^−6^). When the same calculations were performed for Beau-R and M41 infected CK cells, similar results were found with 40% Beau-R infected cell sections containing at least 1 spherule and only 9% M41 infected cell sections (p = 1 × 10^−3^).

These data indicate that the low spherule phenotype of M41 is characteristic of this viral strain and is not dependent on cell type as comparable observations were made between infection of primary CK cells and *ex vivo* TOCs. However, the observations also demonstrate that spherule formation is not associated with viral pathogenicity, and rather is a unique characteristic of the M41 strain. Therefore, this indicates that DMVs, zippered ER and spherules are typical for IBV infection and are neither associated with viral genotype, level of pathogenicity nor laboratory adaptation.

### DMVs and either zippered ER with spherules or convoluted membranes are closely correlated during replication of diverse coronaviruses

**Figure 6:**
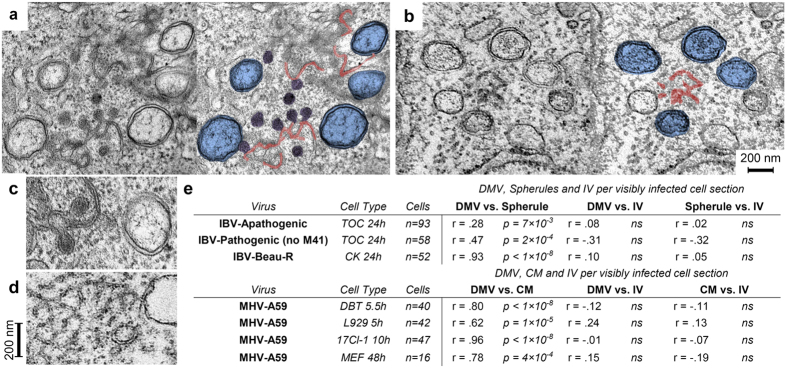
Spatial and temporal correlation between DMVs and other paired membrane structures but not intracellular virions across diverse coronaviruses. Transmission electron micrographs of (**a**) tracheal organ cultures infected with Beau-R and chemically fixed after 24 hours and (**b**) DBT cells infected with MHV-A59 and chemically fixed after 5.5 hours. DMVs (blue), spherules (purple), zippered ER (red in a) and convoluted membranes (red in b) are highlighted. Enlarged images of DMVs and paired membrane structures from (**c**) IBV and (**d**) MHV infected cells. (**e**) Pearson linear correlation coefficients and p values (ns = not significant) for comparisons between the number of DMVs, spherules, convoluted membrane regions and intracellular virions in randomly oriented ultrathin sections through infected cells.

During SARS-CoV infection, DMVs have been shown to appear from 2 hpi with CMs appearing at 3 hpi^10^. Both structures are derived from and connected to the ER, with connections also detectable between CMs and DMV outer membranes. In our previous work, IBV induced membrane rearrangements were not detectable until 7 hpi, when both DMVs and zippered ER with spherules were found. Zippered ER is clearly contiguous with ER and an example of a connection between the ER and a DMV was also identified. However, no connections were observed between IBV induced zippered ER and DMVs. To determine how related these structures are to one another during replication of diverse coronaviruses, in addition to data already described in this study, DBT, L929, 17Cl-I or MEF cells were infected with mouse hepatitis virus (MHV) strain A59. At the indicated times post infection, cells were chemically fixed and analysed by TEM. Examples of IBV Beau-R infected TOCs (Fig. 6a) and MHV-A59 infected DBT cells (Fig. 6b) are shown with additional panels where DMVs are highlighted in blue, spherules are highlighted in purple and zippered ER or CMs highlighted in red. Enlarged images of IBV Beau-R induced structures (Fig. 6c) and MHV-A59 induced structures (Fig. 6d) are also shown for ease of comparison. Finally, the presence of different markers of infection within IBV or MHV-A59 visibly-infected cell sections was calculated (Fig. 6e). Firstly, for IBV infected cells, the presence of DMVs and spherules, DMVs and intracellular virus or spherules and intracellular virus was quantified using data generated in Figs 2 and 4. Samples were categorised into TOCs infected with either apathogenic IBV or pathogenic IBV (excluding M41) and CK cells infected with Beau-R. Data on the number of virus-induced features per cell was used to look for linkages between production of coronavirus DMVs, spherules, convoluted membranes and intracellular virus particles, following the hypothesis that features that were spatially and temporally linked would be more likely to co-occur in ultrathin sections of randomly-oriented cells such as these. Statistically significant correlations were observed between the number of IBV DMVs and spherules per infected cell section, but the number of intracellular virions was not significantly correlated with spherule or DMV abundance (Fig. 6e). The correlation between IBV DMV and spherule abundance was statistically significant in both primary and continuous cells regardless of the pathogenic potential of the strains tested. Similarly, strong correlations were observed between the number of MHV DMVs and convoluted membrane clusters per infected cell, but the number of intracellular MHV virions was not significantly correlated with the abundance of DMVs or convoluted membrane clusters (Fig. 6e). The correlation between MHV DMVs and convoluted membrane abundance was significant for all four conditions tested. Together, these data suggest that both IBV spherules and MHV convoluted membranes commonly co-occur with DMVs in infected cells as part of a double-membrane replicative organelle^24^ that is distinct from the site of virus assembly.

## Discussion

In this study we performed a detailed comparison of the membrane rearrangements induced by a range of different strains of IBV. The selected strains covered a wide variety of viral genotypes, lab adapted, field and vaccine strains as well as pathogenic and apathogenic strains of virus. To our knowledge, this study forms the most comprehensive comparison of membrane rearrangements induced by different virus strains for any coronavirus to date. The ability of each of the selected viruses to replicate in *ex vivo* TOCs was assessed. Indeed, all of the viruses were found to be capable of replication as measured by accumulation of dsRNA in the epithelial cells of the TOC or loss of ciliary activity over the course of infection. All of the strains of IBV inhibited ciliary activity by 96 hpi. Therefore, the ability to inhibit ciliary activity is not associated with the overall pathogenicity of the virus in a bird. Instead, it is more likely, that pathogenicity is linked to the site of virus replication and ability of the virus to spread in the bird. The apathogenic lab adapted and vaccine strains are restricted to sites closer to the site of inoculation and are less capable of spreading to the trachea and other sites within the bird. It was noted, however, that pathogenic field strain 4/91 presented a more gradual inhibition of ciliary activity when compared with the other virus strains, although complete ciliostasis was induced by 96 hpi. It is not possible to titrate this strain by plaque assay in CK cells because this virus does not replicate in CK cells. Therefore, it is possible that the level of infection was lower for this virus. However, the percentage of cells showing signs of virus replication in EM sections and the overall dsRNA accumulation would not indicate that the level of infection was significantly different to any of the other strains of IBV. Therefore, the reason for the difference in the dynamics of inhibition of ciliostasis caused by 4/91 remains unknown.

The ability of each of the selected viruses to induce cellular membrane rearrangements was also determined. When TOCs were infected with each of the virus strains, all three previously identified IBV Beau-R induced membrane structures were identified. This demonstrates that additional, and therefore potentially all, strains of IBV appear to be able to induce zippered ER with associated spherules and DMVs, regardless of viral genotype, lab adaptation or degree of pathogenicity. Upon further assessment, the average diameter of DMVs and spherules was also consistent between the different virus strains. Therefore, the type and size of membrane structures induced by IBV is likely to be a fundamental requirement for virus replication machinery and is not associated with adaptation to grow in embryonated eggs or cell culture and is not associated with pathogenicity. This finding is significant because such a detailed comparison of different strains of coronavirus has not been established. It was previously unknown whether alterations in either efficiency of induction of membrane rearrangements or variations in the types of structure might account for changes in pathogenicity or tropism for cell culture. The results presented here clearly demonstrate that this is not the case.

In our analysis, it was also found that there is a highly significant correlation between the presence of DMVs and spherules within the same IBV infected cell or DMVs and CM within the same MHV infected cell. This indicates that production of DMVs and either zippered ER with spherules or CMs is a linked and possibly simultaneous process. It also demonstrates that the temporal and spatial link between these structures is conserved among the distantly related *Beta-* and *Gammacoronaviruses*. Although no connections were found between zippered ER or spherules with DMVs in our earlier work^14^, when similar experiments were performed in SARS-CoV infected cells, clear connections were found between DMVs, CM and cellular ER^10^. Connections between DMVs and paired ER membranes are also present in cells infected with related equine arterivirus^25^. In addition, studies using recombinant MHV expressing fluorescently-tagged nsp4 demonstrated that the ER is contiguous with virus replication structures^26^. However, although clearly associated in terms of function and presence in infected cells, data suggests that IBV induced DMVs and spherules are discrete structures formed by different mechanisms^14^.

One surprising observation made during this study was that M41 has a low spherule phenotype during replication in both CK cells and TOCs. Although numerous DMVs and virus particles could be detected in M41 infected cells (see Supplementary Fig. 1), very few spherules could be identified. This is despite the fact that virus replication and release of progeny over 96 hours was comparable to Beau-R, consistent with previous work using M41 and Beau-R^19^,^27^. In addition, there was no significant difference between the viruses in the overall levels of gRNA at 8 and 24 hpi. This suggests that RNA synthesis and turnover is comparable for the two viruses. Finally, dsRNA accumulation, indicative of viral RNA synthesis and possibly assembly of replication complexes and formation of DMVs was detectable at 4 hpi in Beau-R infected cells but was found earlier in cells infected with M41, at 3 hpi. These results are consistent with previously published results^14^,^28^. This indicates that, despite the lower number of visible spherules during M41 replication, there is no effect on virus replication. In fact, earlier dsRNA accumulation may indicate that M41 replication complexes assemble earlier after infection of the cell than during Beau-R infection. Interestingly, we have previously reported that recombinant chimeric viruses containing either gene 1 from Beau-R and the structural and accessory genes from M41, or the coding region for nsp15 to the 3′ end from M41 and the remainder of the 5′ end from Beau-R both produce easily detectable spherules^21^. Therefore, the genetic determinants for spherule formation must lie within nsp2 to nsp14. It may be expected that membrane spanning nsps 3, 4 and 6 will play a role in membrane rearrangements, as has been suggested for other coronaviruses^26^,^29^,^30^. However, as other studied coronaviruses do not produce spherules, it cannot be ruled out that further viral proteins may play a role in their formation.

Overall, the data presented here suggests that total spherule number is not associated with level of IBV RNA synthesis and viral fitness, at least in the case of M41. This may be a surprising conclusion because where spherules are induced by other +RNA viruses and are confirmed as sites of viral RNA synthesis, there is a correlation between spherule formation or number and RNA synthesis. Temperature sensitive mutants of Semliki Forest virus (SFV) were found to produce low numbers of spherules at the non-permissive temperature and there was a corresponding reduction in virus replication^31^. Furthermore, inhibition of spherule formation by tomato bushy stunt virus and brome mosaic virus by inhibition of the endosomal sorting complexes required for transport (ESCRT) pathway results in a reduction in viral RNA synthesis^32^,^33^. Finally, viral RNA synthesis and spherule formation are tightly associated during flock house virus and SFV infection as formation of spherules is only triggered by the process of active RNA synthesis^34^,^35^. Therefore, it is likely that the role played by spherules during IBV replication is somewhat different to other +RNA viruses. However, it is notable that the presence of coronavirus induced DMVs is also not tightly correlated with viral RNA synthesis. Although some work has shown that some MHV temperature sensitive mutants have a reduction in DMV formation or aberrant DMV formation at the non-permissive temperature, associated with reduced viral RNA synthesis^36^,^37^, recent work suggests that this is not always the case at permissive temperatures. Changes in DMV number or size were not linked to changes in RNA synthesis^38^ and it was not DMV number and morphology but instead glycosylation of one of the viral nsps that was found to be responsible for the reduction in RNA synthesis for another set of viruses^39^. Clearly the induction of membrane rearrangements is critical for virus replication^40^ and this process is well conserved amongst all +RNA viruses. However, the precise function of the variety of structures induced by coronaviruses remains a complicated story. It is likely that all of the different structures produced each play a role in virus replication and viral RNA synthesis. In this case, where one type of structure is reduced or not present during infection, another structure would be capable of compensating. During replication of IBV and arteriviruses at least, all virus induced structures are comprised of tightly apposed double membranes. It may be that this feature is the important one in terms of viral RNA synthesis and the precise shape of the membrane is less critical.

An alternative explanation is that the total number of spherules required to allow efficient viral RNA synthesis is very low. In this case, the low number of spherules observed during M41 replication would be sufficient to allow replication complex assembly and viral RNA synthesis. The higher number of spherules present in cells infected with other strains of IBV used here would represent an excess, not strictly required for viral RNA synthesis. Indeed, it has long been reported that viral RNA synthesis can be detected within infected cells at very short times post infection^11^,^14^,^41^,^42^,^43^, earlier than any evidence of membrane rearrangements can be observed^10^,^11^,^14^. Perhaps low numbers of membrane rearrangements, below levels of detection by electron microscopy, exist early after infection, but that are sufficient for the levels of RNA synthesis seen at these times. Numerous unanswered questions remain; however, the identification here of two cell culture adapted strains of IBV (Beau-R and M41) that induce variations in membrane rearrangements now provides an exciting opportunity for future work to begin to unravel the complicated story of the mechanism of formation and role of coronavirus induced membrane rearrangements.

## Materials and Methods

### Ethics statement

Primary chick kidney (CK) cells and *ex vivo* tracheal organ cultures (TOCs) were prepared by The Pirbright Institute microbiological services department from chickens produced in the Institute’s poultry production unit. Sacrifice of chickens and embryos was performed by trained staff under a schedule 1 procedure, in accordance with local rules. This procedure does not fall under any UK Home Office licence requirements as procedures were not carried out on live animals. However, studies were carefully considered for animal welfare and ethical implications and were approved via institutional processes. The kidneys were removed from the sacrificed chickens for preparation of the primary CK cells and trachea were removed from the sacrificed chickens or embryos for preparation of *ex vivo* TOCs. All work was performed in a designated establishment, The Pirbright Institute, Compton Laboratory.

### Cells, viruses and antibodies

Primary CK cells were produced from 2–3 week-old specific pathogen free (SPF) Rhode Island Red (RIR) chickens^44^. TOCs were produced from 19 day old SPF embryos^23^,^45^, or 2–3 week-old SPF RIR chickens, using the same procedure. The murine DBT astrocytoma cell line was used to grow the *Betacoronavirus* MHV-A59 as described previously^46^. The apathogenic molecular clone of IBV, Beau-R, has been described previously^20^. The pathogenic lab adapted strain of IBV, described elsewhere as M41-CK but here called M41, is a derivative of M41^47^. The vaccine strain H120 (Bioral, Merial Sanofi) and field isolates 4/91, Italy02 and D1466 (kindly provided by Prof R. Jones and Dr K. Ganapathy, University of Liverpool) were also used. The characteristics of these IBV strains are summarised in Table 1. Anti-tubulin was purchased from Sigma-Aldrich. Anti-dsRNA J2 was purchased from English and Scientific Consulting Bt. Goat anti-mouse secondary antibodies were purchased from Life Technologies.

### Viral growth kinetics in CKCs

CK cells were infected with Beau-R or M41 at an MOI of 0.005. After 1 hour incubation at 37 °C, cells were washed and fresh 1x BES (MEM, 0.3% tryptose phosphate broth, 0.2% bovine serum albumin, 20 mM N,N-Bis(2-hydroxyethyl)-2-aminoethanesulfonic acid (BES), 0.21% sodium bicarbonate, 2 mM L-glutamine, 250 U/ml nystatin, 100 U/ml penicillin, and 100 U/ml streptomycin) added. Cell supernatant was harvested at 1, 24, 48, 72 and 96 hpi. Presence of progeny virus was determined by plaque assay on CK cells.

### Two-step RT-qPCR to analyse viral RNA levels

CK cells were mock infected or infected with either Beau-R or M41 at an MOI of 20. After 1 hour, cells were washed twice with PBS and fresh 1x BES added. Cells were harvested at 2, 8 and 24 hpi using RLT buffer and total RNA extracted using a TissueLyser II (Qiagen) and an RNeasy kit (Qiagen), according to the manufacturer’s protocol. RNA was reverse transcribed using Superscript III (Invitrogen), using 600 ng RNA and primers specific for the 5′ UTR. Quantitative PCR was then performed as described in^14^. Absolute quantitation of cDNA copies was performed using a plasmid standard followed by normalisation against the mock sample from each time point. Finally, the 8 and 24 hpi samples were normalised against input present in the 2 hpi sample.

### Immunofluorescence labelling

CK cells were mock infected or infected with Beau-R or M41 and incubated for 1 hour at 37 °C. Cells were then washed and fresh 1x BES added. Cells were fixed at 3 and 4 hpi using 4% paraformaldehyde in PBS for 30 minutes at room temperature. Cells were then washed once with PBS and permeabilised in 0.1% triton X-100 for 20 minutes. Cells were blocked for 1 hour at room temperature in 0.5% BSA in PBS, and then incubated for 1 hour at room temperature with anti-dsRNA diluted 1:1000 in blocking buffer. After three washes in PBS, cells were incubated for 1 hour at room temperature with goat anti-mouse IgG2a Alexa 568 diluted 1:200 in blocking buffer. Finally cells were washed three times in PBS and nuclei stained with TO-PRO-3 iodide (Life Technologies) diluted in water.

TOCs produced from 19 day old SPF embryos were mock infected or infected with 5 × 10^5^ PFU Beau-R, M41 or H120, or 500 μl D1466, 4/91 or Italy02 grown in SPF eggs, diluted 1:10. TOCs were incubated for 1 hour at 37 °C and fresh medium added. After 24 hours, TOCs were washed with PBS and fixed in 4% paraformaldehyde. TOCs were labelled with anti-dsRNA as described above. Anti-tubulin antibody was diluted 1:1000 and goat anti-mouse IgG1 Alexa 488 was diluted 1:200. Nuclei were stained with 4′,6-diamidino-2-phenylindole (DAPI) diluted in water. Labelled TOCs were mounted onto cavity well microscope slides in mounting medium.

### Transmission electron microscopy of chemically fixed cells

CK cells in 6 well plates were infected with Beau-R or M41 and incubated for 1 hour at 37 °C when fresh 1x BES medium was added. At 24 hpi, cells were washed once in 0.9% saline and scraped into the buffer. Cells were pelleted at 500 × g for 5 minutes at 4 °C and then 500 μl 2% glutaraldehyde in 0.1 M sodium cacodylate. CK cell pellets were dehydrated in increasing concentrations of acetone and then embedded in Agar 100 resin (Agar Scientific). Ultrathin sections that produced a silver interference pattern (50 to 60 nm in thickness) were taken from embedded samples and post- stained with 0.5% uranyl acetate. Electron microscopy was performed at 200 kV on a Philips CM20 equipped with a 2K charge-coupled device (CCD) camera. Cell sections used here each contained a single visible nucleus, with intact nuclear and plasma membranes. Images of DMV, spherule or virion-containing cells meeting these criteria were captured for later analysis. 60–395 cell sections were imaged per sample.

TOCs produced from 19 day old SPF embryos were mock infected or infected with 5 × 10^5^ PFU Beau-R, M41 or H120, or 500 μl D1466, 4/91 or Italy02 grown in SPF eggs, diluted 1:10. TOCs were incubated for 1 hour at 37 °C and fresh medium added. After 24 hours, TOCs were fixed in 2.5% glutaraldehyde, 4% paraformaldehyde in 0.1 M sodium cacodylate for 1 hour at room temperature and then 4 °C overnight. TOCs were then rinsed three times in 0.1 M sodium cacodylate and incubated in 1% osmium tetroxide for 2 hours. After three washes in water, TOCs were incubated in 2% uranium acetate aqueous for 2 hours at 4 °C. TOCs were dehydrated in increasing concentrations of ethanol and then embedded in Agar 100 resin (Agar Scientific). Ultrathin sections with a silver interference pattern (approximately 50 to 60 nm in thickness) were stained with 2% uranyl acetate to enhance contrast. 76–84 cell sections were imaged per sample.

DBT cells were inoculated with 3 PFU of MHV-A59 per cell and maintained at 37 °C. Infected cells were fixed in electron microscopy-grade 4% glutaraldehyde (Sigma) in 0.1 M cacodylate buffer, scraped from the plate, and then postfixed in 0.1 M cacodylate-buffered 1% osmium tetroxide with 1.5% uranyl acetate for 1 h. The cells were dehydrated in increasing concentrations of acetone and then embedded in Agar 100 resin (Agar Scientific). Ultrathin sections that produced a silver interference pattern (50 to 60 nm in thickness) were taken from embedded samples and post- stained with 0.5% uranyl acetate. 205–604 cell sections were imaged per sample. Cell sections used here each contained a single visible nucleus, with intact nuclear and plasma membranes.

### Analysis of membrane structures and statistics

Pixel size for each image was calculated from images of a calibration grid that were recorded at each of the magnifications used to image infected cells. Intracellular features were measured using the boxer module of EMAN^48^ following the procedure previously described for measuring MHV DMVs^38^. The longest and shortest diameters of each DMV, spherule and intracellular virus particle were measured twice each, with a standard deviation of about 2 pixels on average, which equated to approximately 4–10 nm at the level of the specimen. Since most virus-induced intracellular features had elliptical profiles, size was calculated by dividing Ramanujan’s first approximation of perimeter length by π to approximate diameter. Size and shape distributions were compared using Welch’s *t*-test with Bonferroni correction for multiple comparisons. The intracellular abundance of virus-induced features in individual cell-sections was compared using Fisher’s exact test. Linear Pearson correlations between the number of visible DMVs, spherules or intracellular virions in a given cell section were calculated as a way to examine spatial and temporal covariance in IBV-induced features.

## Additional Information

**How to cite this article**: Maier, H. J. *et al.* Extensive coronavirus-induced membrane rearrangements are not a determinant of pathogenicity. *Sci. Rep.* **6**, 27126; doi: 10.1038/srep27126 (2016).

## Supplementary Material

Supplementary Information

## Figures and Tables

**Table 1:** Description of IBV strains used in this study.

Strain	Genotype	Designation	Strain information	Refs
H120	Massachusetts	Vaccine	Nobilis IB H120 vaccine strain produced by serial egg passage - not pathogenic in infected birds	49
Beau-R	Massachusetts	Apathogenic lab	Molecular clone of IBV-Beaudette-CK, which was attenuated by serial egg and CK cell passage - not pathogenic in infected birds	20,50
M41	Massachusetts	Pathogenic lab	Adapted for growth in CK cells by serial passage - severe respiratory symptoms in infected birds	19,47
D1466	D212	Pathogenic field	First isolated in Holland in 1960s - early isolates minimally pathogenic, but recent isolates like this one cause cystic ovaries and nephritis	51,52
4/91	4/91	Pathogenic field	First isolated in UK in 1991 - respiratory symptoms and nephritis	53, 54, 55
Italy02	Italy02	Pathogenic field	First isolated in Italy in 2002 - respiratory symptoms and nephritis in young birds, egg drop in adult birds	56,57

**Table 2:** Quantitation of markers of infection in cells infected with different IBV strains.

*Virus*	*Cell*	*Type*	*Total Cells*	*Visibly infected cell sections*				*Per visibly infected cell section*			*Infected cell sections*	*Infected cell sections with one or more*					
				DMV	Spherule	IV	Any	DMV	Spherule	IV		DMV		Spherule		IV	
H120	*TOC 24 hpi*	V-AP	*n* = *210*	7%	6%	9%	11%	4 ± 1	4 ± 1	15 ± 5	*n* = *24*	63%	–	54%	–	79%	–
Beau-R		L-AP	*n* = *63*	29%	17%	46%	52%	3 ± 1	4 ± 1	18 ± 4	*n* = *69*	58%	*ns*	54%	*ns*	78%	*ns*
M41		L-P	*n* = *395*	8%	1%	12%	13%	2 ± 1	0.1 ± 0.1	19 ± 4	*n* = *53*	62%	*ns*	6%	*p* = *4* × *10*^*−6*^	92%	*ns*
D1466		F-P	*n* = *157*	9%	3%	13%	13%	2 ± 1	1 ± 1	27 ± 5	*n* = *21*	67%	*ns*	24%	*ns*	95%	*ns*
4/91		F-P	*n* = *79*	15%	10%	18%	23%	2 ± 1	2 ± 1	11 ± 4	*n* = *21*	67%	*ns*	48%	*ns*	76%	*ns*
Italy02		F-P	*n* = *60*	10%	5%	10%	18%	3 ± 1	3 ± 2	17 ± 5	*n* = *16*	50%	*ns*	31%	*ns*	63%	*ns*
Beau-R	*CK cell 24 hpi*	L-AP	*n* = *76*	14%	14%	33%	37%	2 ± 1	3 ± 1	35 ± 11	*n* = *52*	42%	–	40%	–	90%	–
M41		L-P	*n* = *84*	19%	2%	37%	39%	1 ± 0.3	0.3 ± 0.2	21 ± 4	*n* = *34*	50%	*ns*	9%	*p* = *1* × *10*^*−3*^	94%	*ns*

V = vaccine, L = lab adapted, F = field, AP = apathogenic, P = pathogenic. The intracellular abundance of virus-induced features in individual cell-sections was compared to H120 using Fisher’s exact test.
