# Supplementary material for: Extensive coronavirus-induced membrane rearrangements are not a determinant of pathogenicity
Source: Sci Rep. 2016 Jun 3;6:27126. doi: 10.1038/srep27126 (PMC4891661; doi:10.1038/srep27126)
Supplement: Supplementary Information [file srep27126-s1.pdf]

Supplementary Figures

Extensive coronavirus-induced membrane rearrangements are not a determinant of pathogenicity

Helena J. Maier[1]*, Benjamin W. Neuman[2], Erica Bickerton[1], Sarah M. Keep[1], Hasan Alrashedi[2], Ross H. Hall[1] and Paul Britton[1#]

[1]The Pirbright Institute, Pirbright, Surrey, UK; [2]School of Biological Sciences, University of Reading, Reading, Berkshire, UK

[#]now retired

*Address correspondence to Helena J. Maier helena.maier@pirbright.ac.uk

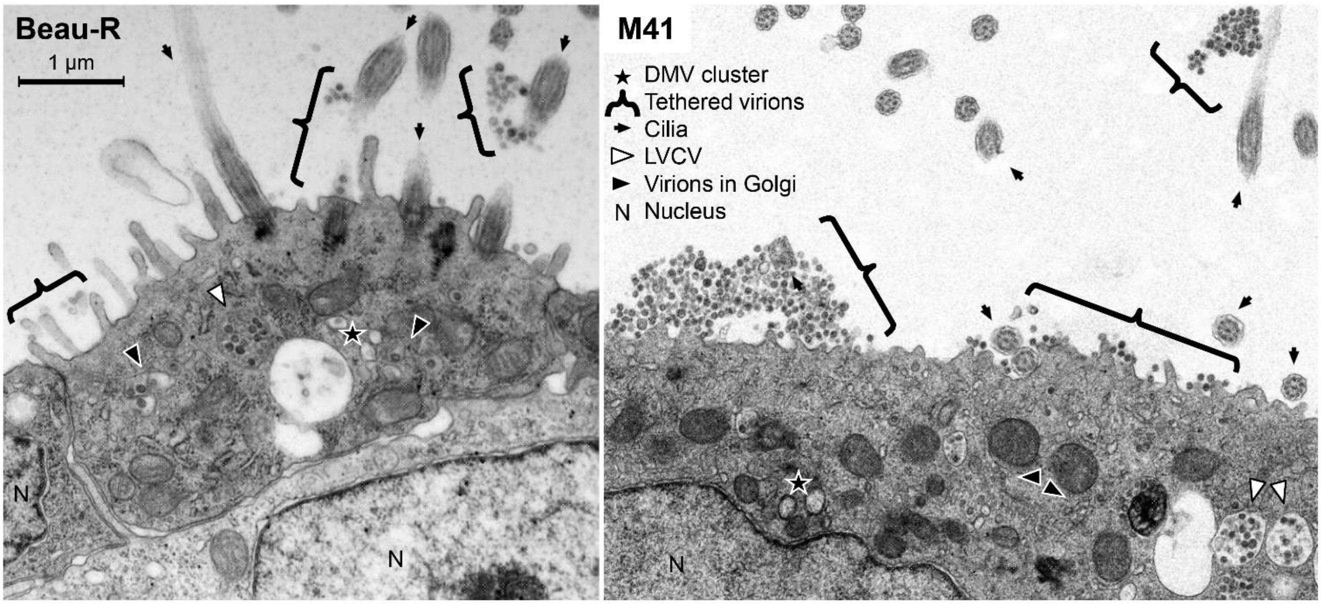

Supplementary Figure S1. Overview of markers of virus infection. Tracheal organ culture (TOCs) infected with Beau-R or M41. After 24 hrs, TOCs were fixed an imaged by TEM. Cells were imaged on a low magnification to indicate the extent of markers of virus infection.
